# Surgery

**Published:** 1893-04

**Authors:** 


### *SELECTIONS AND ABSTRACTS.*

---

### SURGERY.

---

EDITED BY DR. FLOYD W. McRAE.

---

### BLOODLESS AMPUTATION AT THE HIP-JOINT.

Dr. N. Senn describes an improved method of performing bloodless amputation at the hip-joint (Chicago *Clin. Rev.*). The paper is illustrated with a number of photo-engravings representing different stages of the operation. The following conclusions represent the principal points in the method :

1. Preliminary dislocation of the head of the femur and clearing the shaft of this bone of all soft tissues down to the proposed line of amputation through an external straight incision requires less time, is attended by less hemorrhage and shock than when this part of the operation is done after circular amputation, as advised by von Esmarch and others.

2. The external straight incision is the same as von Langenbeck's incision for resection of the hip-joint, differing only in length.

3. After dislocation of the femur the soft tissues are tunneled with a hemostatic forceps, which is entered through the external wound on a level with the trochanter minor to a point on the inner aspect of the thigh behind the adductor muscles and about two inches below the ramus of the ischium where a counter opening two inches in length is made.

4. Bloodless condition of the limb should be secured by elastic compression or vertical position prior to tying the elastic constrictors.

5. An elastic tube three-quarters of an inch in diameter and about four feet in length is grasped with the forceps in the center and drawn through the tunnel made by the forceps.

6. After dividing the elastic tube in the center the base of the thigh is constricted by drawing firmly and tying the anterior constrictor in front of the anterior section, while the posterior constrictor, after being drawn tight behind the posterior section the two ends are crossed, and then made to encircle the whole thigh, when the ends are again drawn firm and tied or otherwise secured above the anterior constrictor.

7. A long and a short oval cutaneous flap should invariably be made in all amputations at the hip-joint.

8. In preference a long anterior and a short posterior flap should be selected.

9. The transverse section through the muscles should be somewhat conical in shape, the apex of the cone corresponding to the tunnel made by enucleation of the upper portion of the shaft of the femur.

10. Resection of the end of the sciatic nerve and ligation of all vessels that can be found should be done before the removal of the constrictors.

11. The femoral arteries should be secured by a double catgut ligature half an inch apart, the one on the proximal side including also the accompanying vein.

12. The posterior constrictor should be removed first, and all hemorrhage arrested by ligation and compression before the anterior constrictor is removed.

13. The upper part of the wound corresponding to the acetabulum should be drained with an iodoform gauze tampon, and the remaining part of the wound by one or more tubular drains. —*Medical Review.*

---

Cripps (Harrison). "The Treatment of Complete Obstruction of the Large Intestines by Temporary Typhlotomy."—*British Medical Journal*, February 23, 1893.

Dr. Cripps, after outlining the difference in the symptoms of obstruction of the large intestines and obstruction of the small intestines, recommends first, in all cases of obstruction of the large intestine, the use of injections of warm water through a half inch rubber tube passed as far into the bowel as it will go without the slightest force. The injections are persisted in as long as there is discoloration of the liquid, but suspended as soon as it returns clear.

He advises that incision first be made on left side for following reasons:

1. "Because in the majority of cases such an incision will bring the operator at once to the site of obstruction."

2. "If the obstruction is of such a nature that necessitates colotomy, the exploratory incision serves as a means of completing the operation."

3. "If the obstruction is not found at the site expected, the emptied sigmoid or descending colon at once shows that it is higher up and will almost certainly have to be dealt with by a temporary typhlotomy."

4. "It must be remembered that in most cases the whole abdomen is tightly distended by inflated intestine, and that it would often be impracticable to drag any part of the ascending or descending colon to an opening in the middle line without undue tension."

This is best done by an incision three inches from the anterior superior iliac spine, bisecting an imaginary line drawn from anterior superior iliac spine to umbilicus. The subsequent steps of the operation are determined by the conditions presenting. If the conditions are such as not to be satisfactorily dealt with through this incision the wound is immediately closed and another incision made over the cæcum. A small portion of the cæcum is immediately stitched to the abdominal parieties. A small opening is then made in cæcum) preferably through trocar and canula with tube attached), allowing escape of gas and liquid feces without soiling the wound. The patient's life is saved and the case may be considered leisurely, either finally closing the fecal fistula or establishing a permanent opening as the case may necessitate.

Two cases are reported in exemplification.

BULL (WM. T.). "Chronic Relapsing Appendicitis."—*New York Med. Record*, March 18, 1893.

Dr. Bull reported, in tabular form, twelve operations done during quiescence—eleven successful, one fatal. He draws the distinction between "recurrent appendicitis" and "chronic relapsing appendicitis" as described by Talamon. In the former, the attacks are irregular, recurring at long intervals, followed by apparent complete recovery, each attack predisposing to, but independent of, subsequent attacks. In the latter there is no return to health; there are pains and soreness in right iliac fossa with usually coexistence of inflammatory tumor subject to frequent inflammatory exacerbations. Operation is not advised in recurrent appendicitis. Recommends operation during quiescence in chronic relapsing form. There is less danger from infection, a smaller incision is required and more time is allowed for preparation during quiescence. He reports statistics of seventy-six cases including his twelve with one death. He recommends the oblique incision and inversion of the stump of the appendix.

---

### SUCCESSFUL SPLENECTOMY FOR WANDERING SPLEEN.

At a meeting of the Clinical Society of London, Bland Sutton (*British Medical Jounaal*) reported the case of a woman, twenty-two years old, who had become cognizant of the presence of a movable swelling in the lefi half of the abdomen, which became the seat of acute pain, with which were associated vomiting and diarrhœa. An exploratory operation showed the tumor to be a greatly enlarged, movable spleen, with a twisted pedicle. The pedicle was untwisted and the organ returned to the left hypochondrium. The woman did quite well for some time, but after the lapse of more than three months the swelling reappeared and the patient was suddenly seized with acute abdominal pain, vomiting, diarrhœa, and hemorrhage from the vagina. The spleen at different times found its way into the most various situations, so that finally it was determined to perform splenectomy. The removed organ weighed sixteen ounces; its texture was normal. Recovery ensued without complication. No difference was observed between the number and proportion of blood-corpuscles before the operation and those after operation.—*Medical News*.

